# Circulating monocyte populations as biomarker for abdominal aortic aneurysms: a single-center retrospective cohort study

**DOI:** 10.3389/fimmu.2024.1418625

**Published:** 2024-07-30

**Authors:** Johannes Klopf, Branislav Zagrapan, Annika Brandau, Peter Lechenauer, Catharina J. Candussi, Patrick Rossi, Nihan Dide Celem, Michael Ziegler, Lukas Fuchs, Hubert Hayden, Claus G. Krenn, Wolf Eilenberg, Christoph Neumayer, Christine Brostjan

**Affiliations:** ^1^ Division of Vascular Surgery, Department of General Surgery, University Hospital Vienna, Medical University of Vienna, Vienna, Austria; ^2^ Intensive Care Medicine and Pain Medicine, Department of Anesthesia, University Hospital Vienna, Medical University of Vienna, Vienna, Austria

**Keywords:** abdominal aortic aneurysm, biomarker, diagnosis, intermediate monocytes, monocyte-platelet aggregates, prognosis

## Abstract

**Background:**

Abdominal aortic aneurysm (AAA) development is driven by inflammation, in particular myeloid cells, which represent attractive biomarker candidates. Yet to date, the maximum aortic diameter is the only clinically applied predictor of AAA progression and indicator for surgical repair. We postulated that aortic inflammation is reflected in a systemic change of monocyte populations, which we investigated regarding marker potential in AAA diagnosis and prognosis.

**Methods:**

We conducted a single-center retrospective cohort study in a diagnostic setting, measuring monocyte subsets by flow cytometry in peripheral blood samples of 47 AAA patients under surveillance, matched with 25 healthy controls and 25 patients with peripheral artery disease (PAD). In a prognostic setting, we acquired longitudinal data of 60 AAA patients including aneurysm growth assessment by computed tomography at 6-month intervals.

**Results:**

Blood levels of total monocytes, CD16^+^ monocytes and particularly intermediate monocytes were significantly increased in AAA patients versus healthy individuals and were also elevated compared to PAD patients. The combination of intermediate monocyte and D-dimer blood levels outperformed the individual diagnostic marker values. Additionally, the elevated concentrations of total monocytes, intermediate monocytes, and monocyte-platelet aggregates (MPA) were suited to predict rapid AAA progression over short-term periods of six months. Of note, MPA were identified as independent predictor of AAA disease progression in multivariable analysis.

**Conclusion:**

Circulating monocyte subsets are elevated in AAA patients and support diagnosis and prediction of aneurysm progression. Monocyte subsets and D-dimer reflect different hallmarks (inflammation and hemostasis) of AAA pathology and when combined, may serve as improved biomarker.

## 1 Introduction

An abdominal aortic aneurysm (AAA) is diagnosed when a full-thickness dilatation of the abdominal aorta reaches ≥30 mm (1, 2). Histologically, the AAA wall is characterized by infiltration of immune cells, loss of elastic fibers and smooth muscle cell plasticity and death (3–5). Risk factors for AAA are male sex, advanced age, smoking, positive family history and presence of other cardiovascular diseases such as hypertension, hyperlipidemia, ischemic heart disease or peripheral artery disease (PAD) (6, 7). While mostly asymptomatic, AAA is characterized by continuous disease progression and rupture risk associated with a high death rate (8–10). To date, there is a lack of pharmaceutical treatment options to control aneurysm growth, and the maximum aortic diameter is the only clinically approved predictor of AAA progression and indicator for surgical repair (1, 3). However, individual risk of progression or rupture is not always accurately represented by aneurysm diameter alone (1, 2). Therefore, the search for diagnostic and prognostic biomarkers is imperative as AAA is a primary cause of cardiovascular mortality. Among blood-based AAA biomarkers, plasma D-dimer was reported to be associated with AAA presence and positively correlated with AAA diameter, intraluminal thrombus (ILT) size and AAA growth (11–17). Considering AAA etiology, not only the process of hemostasis/fibrinolysis but also the accumulation and activation of inflammatory cells such as distinct monocyte populations, are of particular biomarker interest, as alterations may reflect AAA disease state and progression (18).

Chronic inflammation in adventitia and ILT is central in AAA pathogenesis: preclinical and clinical studies link multifactorial inflammatory processes such as immune cell infiltration, extracellular matrix degradation, release of proteases and cytokines as well as generation of reactive oxygen species (ROS) with the pathological weakening and dilatation of the aortic wall (3, 19). The essential contribution of monocytes to these processes was demonstrated in mouse models where monocyte- or macrophage-released proteases were found to drive matrix destruction in AAA formation (20). Furthermore, monocytes/macrophages were documented as a central source of cytokines and chemokines propagating the local inflammatory reaction at the aneurysm site (18).

In contrast to soluble factors, phenotypic alterations of leukocytes, also of monocyte populations, are largely unexplored in the context of AAA (21, 22). In humans, two cell surface markers, CD14 and CD16, serve to distinguish the three main subsets of monocytes (23–26). Based on their expression levels, monocytes can be divided into classical (CD14^++^CD16^-^), intermediate (CD14^++^CD16^+^), and non-classical monocytes (CD14^+^CD16^++^) (25, 27, 28). Classical monocytes are known for antimicrobial defense mechanisms such as phagocytosis (25, 27, 29). Non-classical monocytes are strong producers of cytokines and have the property of vascular crawling and patrolling (30). Intermediate monocytes engage in antigen presentation and tissue remodeling, but may also show high ROS production in inflammatory environments (27, 31). Notably, increased levels of intermediate monocytes are associated with a higher risk of cardiovascular events (32). A previous study demonstrated a decrease of classical and an increase of intermediate monocytes in patients with AAA (21), while we found an overall increase in circulating CD16^+^ monocytes in AAA patients compared to healthy controls (22). Data on the non-classical monocytes in AAA are controversial (21, 22, 33).

Platelets are also significant participants at sites of vascular inflammation, and enhanced monocyte interactions with platelets have been found in cardiovascular and inflammatory diseases (34–38). Of note, intermediate and non-classical monocytes show the highest tendency to form monocyte-platelet aggregates (MPA), and significantly elevated levels represent a risk factor for thrombotic diseases (35, 39).

To date, there are no studies investigating the prognostic biomarker potential of circulating monocyte subsets in human AAA. Given the importance of monocytes in AAA pathogenesis, we postulated that chronic aortic inflammation is reflected in a systemic change in monocyte cell populations rather than being limited to the local AAA site. Here, we aimed to investigate the diagnostic and prognostic value of monocyte subsets for the AAA disease state as well as aneurysm progression. We hypothesized that levels of distinct monocyte subsets are elevated in AAA patients (not only compared to healthy individuals but also to patients with other cardiovascular conditions such as PAD), that they reflect advanced AAA disease state, and predict the rate of AAA progression.

## 2 Methods

### 2.1 Study population

All studies involving human subjects were approved by the institutional ethics committee of the Medical University of Vienna (license no. 1729/2014) and conducted in accordance with The Code of Ethics of the World Medical Association (Declaration of Helsinki). The STROCSS 2021 and STROBE Guidelines (40, 41) have been followed in reporting this single-center retrospective cohort study which was retrospectively registered with the Research Registry and the unique identifying number: researchregistry7647. Written informed consent was obtained from all participants. AAA patients were recruited, diagnosed, and monitored at the outpatient clinic of the tertiary University Hospital Vienna (Division of Vascular Surgery, Department of General Surgery, Medical University of Vienna, Austria) between 2014 and 2021. Healthy and PAD control groups were recruited within the same time frame from general surgery, urology, angiology, and ophthalmology patients (appearing for routine check-ups). Demographics of study participants were recorded by a structured questionnaire. Patient and control data/samples were collected prospectively in the course of a previous study and then analyzed retrospectively for this study.

**Figure 1:**
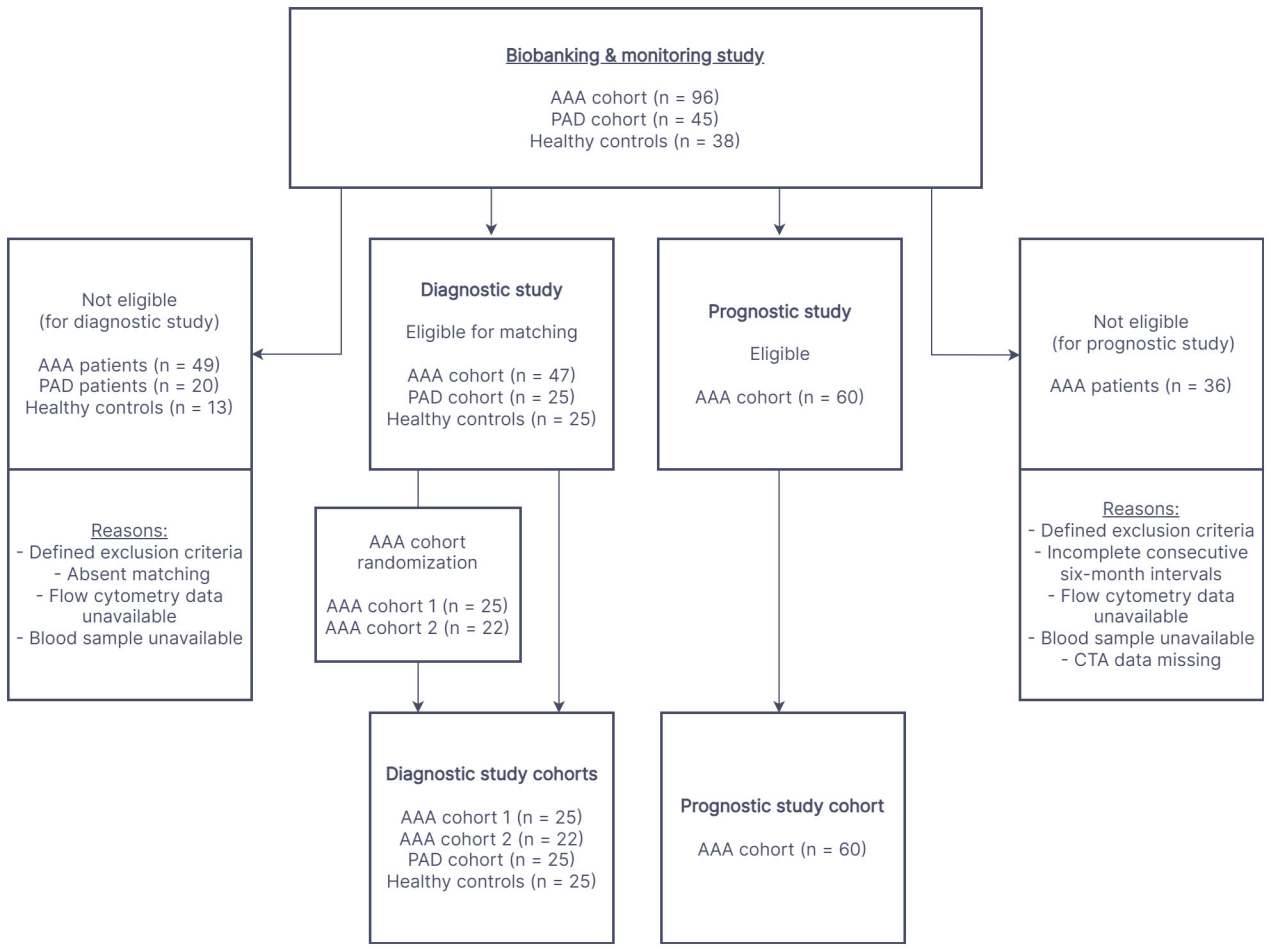
The flow chart illustrates the process of participant recruitment for the diagnostic and prognostic study. The cohorts in both study settings were derived from a prospective biobanking and monitoring study. AAA, abdominal aortic aneurysm; PAD, peripheral artery disease; CTA, computed tomography angiography.

**Figure 1** depicts the enrollment procedure for study participants. For the diagnostic evaluation, data collected from 47 AAA patients (divided in two AAA cohorts for 1:1 matching, AAA patient cohort 1, n=25 and AAA patient cohort 2, n=22) without indication for surgery or prior to elective repair were compared to the control groups of 25 healthy controls and 25 PAD patients. The four cohorts were matched one-to-one in age (within two years), sex and if possible, also in smoking habit. Furthermore, the AAA and PAD cohorts were matched 1:1 for clinically manifest cardiovascular disease (composite of: previous myocardial infarction, angina pectoris or cerebrovascular disease). In the prognostic setting, a cohort of 60 AAA patients (derived from the continued biobanking and monitoring study) was longitudinally observed and analyzed at six-month intervals. In total, 198 monitoring periods of six-months and 148 monitoring periods of twelve-months AAA progression were analyzed.

Recent (< 12 months) malignancy or chemotherapy, hematological or systemic autoimmune disease and organ transplantation were exclusion criteria for all participants. Any aneurysmal disease of the aorta or lower extremity arteries was an exclusion criterion for participants in the PAD or healthy control groups, as confirmed by ultrasound analysis at the time of study inclusion or by an available recent (≤ 6 months) imaging record covering the abdominal vessels. Since healthy participants were age-matched, chronic conditions frequent in advanced age (such as hypertension, hyperlipidemia or diabetes) were not considered an indication for exclusion. Yet, clinically manifest cardiovascular disease in terms of acute events and interventions (myocardial infarction, vascular stents, coronary artery bypass grafts) or clinically documented PAD and coronary heart disease were applied as exclusion criterion for the healthy control cohort.

### 2.2 Flow cytometry of monocyte populations

Detection of monocyte subsets was based on whole blood immunofluorescent staining with antibodies against HLA-DR, CD14, CD16, CD41 and flow cytometry as detailed in the **Supplementary Material File**: **Supplementary Methods** and **Supplementary Figure 1**.

### 2.3 Morphometric AAA analyses

AAA patients underwent computed tomography angiography (CTA) scans at study inclusion and at repeated six-months follow-up visits for the prognostic study as previously described by our group (10, 42, 43). Morphometric AAA analyses were performed with syngo.via (version VB40B, Siemens Healthineers, Forchheim, Germany) and impax EE (Agfa-Gevaert, 17 Mortsel, Belgium) imaging software. Measurements of maximum AAA diameter (and aortic segment volume) were completed by one of two trained radiologic experts, with a documented mean intraobserver variability of 0.14 mm and interobserver variability of 0.20 mm (10, 42, 43).

### 2.4 Statistical analysis

For patient characteristics, median and interquartile range (IQR) were calculated for continuous variables, absolute and relative frequencies for categorical variables. Based on the Kolmogorov–Smirnov test, the majority of acquired data (investigated parameters) were not distributed normally. Hence, non-parametric tests were employed for group comparisons of continuous variables by Wilcoxon signed rank test for paired samples or Mann-Whitney U test (Wilcoxon rank sum test) for independent samples, and by Spearman test for parameter correlations. In addition, for categorical variables, χ² or Fisher exact test were applied. Since this was an explorative study, no adjustments for multiplicity of errors were made. Of note, global hypothesis tests for >2 groups such as ANOVA or Kruskal-Wallis test (which include a limited form of multiplicity adjustment) were not conducted, as the main focus of the study was on the particular pair-wise comparison of 1:1 matched cohorts and the family-wise control of the type I error rate is in general not considered a necessity for exploratory studies. The diagnostic marker potential was characterized by receiver operating characteristic (ROC) analysis and the area under the ROC curve (AUC). Slow versus fast AAA disease progression was defined as < 2 mm or ≥ 2 mm increase in maximum AAA diameter over the next six months and < 4 mm or ≥ 4 mm increase over the next twelve months, reflecting previously published cut-offs (44). Univariable and multivariable binary logistic regression was further conducted to assess the prognostic parameter potential to predict fast AAA growth over the next 6 months. The multivariable regression model for predicting fast AAA growth included the absolute blood level of monocyte-platelet aggregates and as co-variates the maximum AAA diameter at baseline, patient sex and age, smoking behavior, and comorbidities such as hypertension and hyperlipidemia. Two-sided p-values below 0.05 were considered statistically significant. Please note that the terms “statistical trend” or “borderline difference” were used for p-values between 0.05 and 0.1 to indicate potential data trends which were, however, not statistically significant for the current study groups. The data analysis was conducted with SPSS (version 27.0, SPSS Inc., Chicago, Illinois, U.S.A.) and GraphPad Prism (version 8.0.2, GraphPad Software Inc., San Diego, California, U.S.A.).

## 3 Results

### 3.1 Demographic differences of patient and control collectives

Study participants were predominantly male (92–95.5% men in each cohort). Regarding the matching criteria, the cohorts were well comparable (**Table 1**). Yet, despite a very close age range, a significant difference was recorded between the age distribution of AAA cohort 1 versus healthy controls with median (IQR) values of 67.6 (11) and 67.3 (11) years, respectively (*p* = 0.009). AAA patient cohort 1 (n=25) and AAA patient cohort 2 (n=22) did not show significant differences in AAA maximum diameter, aortic segment volume and associated ILT size as well as AAA family history. No significant variations in systolic and diastolic blood pressure were identified between the groups, except for an increased mean systolic blood pressure in PAD patients compared to healthy controls. Risk factors related to cardiovascular diseases such as smoking and hyperlipidemia were more prevalent in patients with AAA and PAD compared to healthy individuals, in line with a higher frequency of coronary heart disease. In addition, patients of AAA cohort 1 and PAD patients were significantly more often affected by hypertension and diabetes mellitus type 2 compared to healthy controls. Of note, AAA patient cohort 2 was more affected by chronic obstructive pulmonary disease than any other study group. With regard to other comorbidities and cardiovascular events such as myocardial infarction and stroke, no significant variabilities were observed between cohorts. However, compared to healthy controls, the diseased groups differed significantly by an up to six-fold higher rate of antiplatelet, antihypertensive, and lipid-lowering therapy.

**Table 1:** Demographics of AAA patients and control groups in the diagnostic study.

*Parameter*		n	*Healthy* *(n=25)*	*AAA cohort 1* *(n=25)*	*AAA cohort 2* *(n=22)*	*PAD* *(n=25)*	*p value*
*Metric variables*			Median (IQR)	Median (IQR)	Median (IQR)	Median (IQR)	
*Age [years]*		25/25/22/25	67.3 (11)	67.6 (11)	66.3 (10)	68.4 (11)	**H:A1 – 0.009 ^a^**
*Body mass index [kg/m²]*		25/24/22/25	27.0 (5.0)	28.1 (6.9)	27.5 (6.3)	25.5 (3.0)	**A1:P – 0.018 ^a^**
*Smoking pack-years [py]*		17/22/20/23	20.0 (33.0)	45.0 (25.8)	47.5 (23.1)	37.0 (33.0)	**H:A2 – 0.033 ^a^**
*Mean systolic blood pressure [mmHg]*		25/24/19/25	130.0 (15.0)	131.7 (17.0)	130.0 (12.0)	143.0 (33.0)	**H:P – 0.004 ^a^**
*Mean diastolic blood pressure [mmHg]*		25/24/19/25	80.0 (10.0)	80.0 (12.0)	80.0 (16.0)	71.0 (22.0)	n.s. ^a^
*Maximum (AAA diameter [mm]*		24/20		52.7 (17.5)	48.4 (11.1)		0.546 ^a^
*Aortic segment volume [cm³]*		19/19		114.2 (87.4)	96.3 (83.9)		0.256 ^a^
*Maximum ILT diameter [mm]*		21/19		13.8 (15.2)	14.5 (11.0)		0.705 ^a^
*ILT volume [cm³]*		19/19		62.9 (80.5)	27.5 (55.9)		0.256 ^a^
*Categorical variables*			n (%)	n (%)	n (%)	n (%)	
*Sex*	*Men*		23 (92.0)	23 (92.0)	21 (95.5)	23 (92.0)	0.959 ^b^
	*Women*		2 (8.0)	2 (8.0)	1 (4.5)	2 (8.0)	
*Smoking*	*Never*		8 (32.0)	2 (8.0)	0 (0)	2 (8.0)	**H:A1 – 0.034 ^b^** **H:A2 – 0.010 ^b^** **H:P – 0.034 ^b^**
	*Past*		13 (52.0)	11 (44.0)	9 (40.9)	15 (60.0)	0.090 ^b^
	*Current*		4 (16.0)	12 (48.0)	12 (54.5)	8 (32.0)	**H:A1 – 0.015 ^b^** **H:A2 – 0.008 ^b^**
*AAA family history*			2 (8.0)	2 (8.0)	4 (18.2)	2 (8.0)	0.352 ^b^
*Hypertension*			13 (52.0)	24 (96.0)	16 (72.7)	23 (92.0)	**H:A1 – < 0.001 ^b^** **H:P – 0.002 ^b^** **A1:A2 – 0.040 ^c^**
*Hyperlipidemia*			7 (28.0)	21 (84.0)	17 (77.3)	19 (76.0)	**H:A1 – < 0.001 ^b^** **H:A2 – 0.001 ^b^** **H:P – 0.001 ^b^**
*Coronary heart disease*			0 (0)	11 (44.0)	8 (36.4)	9 (36.0)	**H:A1 – < 0.001 ^b^** **H:A2 – 0.001 ^c^** **H:P – 0.002 ^c^**
*Myocardial infarction*			0 (0)	6 (24.0)	5 (22.7)	4 (16.0)	0.125 ^b^
*Stroke*			1 (4.0)	3 (12.0)	1 (4.5)	5 (20.0)	0.214 ^b^
*Diabetes mellitus type 2*			3 (12.0)	13 (52.0)	3 (13.6)	9 (36.0)	**H:A1 – 0.002 ^b^** **H:P – 0.047 ^b^** **A1:A2 – 0.006 ^b^**
*PAD*			0 (0.0)	1 (4.0)	2 (9.1)	25 (100.0)	**H:P - < 0.001 ^c^** **P:A1 - < 0.001 ^b^** **P:A2 - < 0.001 ^b^**
*COPD*			1 (4.0)	4 (16.0)	11 (50.0)	5 (20.0)	**H:A2 – < 0.001 ^b^** **A1:A2 – 0.013 ^b^** **A2:P – 0.030 ^b^**
*Antiplatelet therapy*			5 (20.0)	22 (88.0)	18 (81.8)	21 (84.0)	**H:A1 – < 0.001 ^b^** **H:A2 – < 0.001 ^b^** **H:P – < 0.001 ^b^**
*Parameter*		n	*Healthy* *(n=25)*	*AAA cohort 1* *(n=25)*	*AAA cohort 2* *(n=22)*	*PAD* *(n=25)*	*p value*
*Categorical variables*			n (%)	n (%)	n (%)	n (%)	
*Anticoagulation therapy*			2 (8.0)	3 (12.0)	4 (18.2)	4 (16.0)	0.741 ^b^
*Antihypertensive therapy*			13 (52.0)	24 (96.0)	19 (86.4)	20 (80.0)	**H:A1 – < 0.001 ^b^** **H:A2 – 0.012 ^b^** **HC:P – 0.037 ^b^**
*Lipid-lowering agents*			4 (16.0)	23 (92.0)	17 (77.3)	23 (92.0)	**H:A1 – < 0.001 ^b^** **H:A2 – < 0.001 ^b^** **H:P – < 0.001 ^b^**

A1, AAA patient cohort 1; A2, AAA patient cohort 2; AAA, abdominal aortic aneurysm; COPD, chronic obstructive pulmonary disease; H, healthy control cohort; ILT, intraluminal thrombus; IQR, interquartile range; P, PAD patient cohort; PAD, peripheral artery disease; n, number of individuals; n.s., not significant. Values are given as median with IQR for continuous variables, or n (%) for categorical variables. The following statistical tests were applied: ^a^ Wilcoxon signed rank test, ^b^ χ² test, ^c^ Fisher exact test. For pairwise group comparisons, only significant p values are listed and highlighted in bold.

In the prognostic study design, 60 AAA patients without indication for surgical repair were followed at 6-month intervals (median 5.98 months ± 0.69 IQR) for observation of AAA expansion. In total, this comprises 198 analyzed monitoring periods of six-months and 148 monitoring periods of twelve-months AAA progression. Ten women (16.7%) and 50 men (83.3%) were included in the prognostic study (**Table 2**). In median, the age of the AAA patients was 71.0 years at study entry, the body mass index indicated overweight with a value of 27.5 kg/m^2^, and a median of 42.5 pack-years reflected the high prevalence of smoking behavior. The baseline CTA analysis revealed a median maximum aneurysm diameter of 45.1 mm and a corresponding aortic segment volume of 66.5 cm³. Hypertension (88.3%) and hyperlipidemia (83.3%) were the most frequent comorbidities among AAA patients, which was also reflected in the commonly prescribed antihypertensive agents (85.0%), antiplatelet medication (90.0%) and lipid-lowering agents (91.7%) in this prognostic study collective.

**Table 2:** Demographics of AAA patients in the prognostic study.

*Parameter*		n	*AAA cohort (n=60)*
*Metric variables*			Median (IQR)
*Age [years] at first visit*		60	71.0 (11.0)
*Body mass index [kg/m²] at first visit*		60	27.5 (6.3)
*Smoking pack-years [py] at first visit*		56	42.5 (28.8)
*Mean systolic blood pressure [mmHg]*		59	131.3 (20.0)
*Mean diastolic blood pressure [mmHg]*		59	80.0 (12.0)
*Maximum AAA diameter [mm] at first visit*		55	45.1 (11.1)
*Aortic segment volume [cm³] at first visit*		44	66.5 (52.5)
*Maximum ILT diameter [mm] at first visit*		50	11.5 (12.0)
*ILT volume [cm³] at first visit*		45	24.9 (37.6)
*Categorical variables*			n (%)
*Sex*	*Women*		10 (16.7)
	*Men*		50 (83.3)
*Smoking*	*Never*		4 (6.7)
	*Past*		33 (55.0)
	*Current*		23 (38.3)
*AAA family history*			5 (8.3)
*Hypertension*			53 (88.3)
*Hyperlipidemia*			50 (83.3)
*Coronary heart disease*			24 (40.0)
*Myocardial infarction*			13 (21.7)
*Stroke*			4 (6.7)
*Diabetes mellitus type 2*			19 (31.7)
*Parameter*		n	*AAA cohort (n=60)*
*Categorical variables*			n (%)
*PAD*			18 (30.0)
*COPD*			15 (25.0)
*Antiplatelet therapy*			54 (90.0)
*Anticoagulation therapy*			11 (18.3)
*Antihypertensive therapy*			51 (85.0)
*Lipid-lowering agents*			55 (91.7)

AAA, abdominal aortic aneurysm; COPD, chronic obstructive pulmonary disease; ILT, intraluminal thrombus; IQR, interquartile range; n, number of individuals. Values are given as median with IQR for continuous variables, or n (%) for categorical variables.

### 3.2 Circulating monocyte populations are increased in AAA

In the diagnostic comparative analysis of circulating monocytes (**Table 3**), the total monocyte count was significantly elevated in AAA patient cohort 2 compared to the healthy control collective (579 vs 488 N/μl, *p* = 0.024). Yet, there was only a trend to higher monocyte counts in AAA patient cohort 1 (531 vs 488 N/μl, *p* = 0.211). Patients with PAD had no significant difference in total circulating monocytes when compared to AAA patients, but also showed a statistical trend for elevated levels in comparison to the healthy control collective (528 vs 488 N/μl, *p* = 0.088).

**Table 3:** Comparison of circulating monocyte subsets and monocyte-platelet aggregates as well as D-dimer between AAA patients and control groups.

*Parameter*	n	*Healthy* *(n=25)*	*AAA cohort 1* *(n=25)*	*AAA cohort 2* *(n=22)*	*PAD* *(n=25)*	*p value*
		Median (IQR)	Median (IQR)	Median (IQR)	Median (IQR)	
*Total monocytes* *[N/μl]*	25/25/22/25	488 (250)	531 (262)	579 (437)	528 (163)	H:A2 – **0.024** H:P – 0.088 A1:A2 – 0.062
*Classical monocytes* *[N/μl]*	25/25/22/25	396 (197)	390 (268)	430 (433)	418 (158)	H:A2 – **0.020** A1:A2 – 0.095
*Intermediate monocytes* *[N/μl]*	25/25/22/25	32 (30)	56 (43)	53 (41)	46 (29)	H:A1 – **0.006** H:A2 – **0.011**
*Non-classical monocytes* *[N/μl]*	25/25/22/25	43 (63)	58 (44)	62 (63)	69 (61)	n.s.
*CD16^+^ monocytes* *[N/μl]*	25/25/22/25	75 (79)	130 (90)	115 (95)	112 (88)	H:A1 – **0.037** H:A2 – 0.067
*Monocyte-platelet aggregates* *[N/μl]*	25/25/22/25	74 (62)	67 (64)	86 (76)	69 (66)	n.s.
*Total monocytes* *[% of leukocytes]*	25/25/22/25	8.80 (4.30)	8.80 (4.00)	9.35 (3.73)	7.50 (1.20)	H:P – 0.095 P:A2 – **0.042**
*Classical monocytes* *[% of monocytes]*	25/25/22/25	81.53 (10.58)	76.61 (12.84)	81.35 (10.07)	77.28 (11.60)	H:A1 – 0.074
*Intermediate monocytes* *[% of monocytes]*	25/25/22/25	6.99 (3.49)	9.43 (5.08)	8.21 (3.28)	7.21 (5.23)	H:A1 – **0.004** H:A2 – 0.095 P:A1 – **0.048**
*Non-classical monocytes* *[% of monocytes]*	25/25/22/25	11.69 (9.71)	10.80 (9.08)	9.80 (6.94)	11.13 (9.11)	n.s.
*CD16^+^ monocytes* *[% of monocytes]*	25/25/22/25	18.47 (10.58)	23.39 (12.84)	18.65 (10.07)	22.72 (11.60)	H:A1 – 0.074
*Monocyte-platelet aggregates* *[% of monocytes]*	25/25/22/25	13.36 (6.10)	12.42 (7.52)	14.42 (12.92)	10.77 (11.35)	n.s.
*D-dimer* *[μg/ml]*	24/21/19/24	0.43 (0.44)	1.00 (0.96)	0.60 (1.18)	0.56 (0.55)	H:A1 – **0.005** H:A2 – **0.012** P:A1 – 0.052

A1, AAA patient cohort 1; A2, AAA patient cohort 2; AAA, abdominal aortic aneurysm; COPD, chronic obstructive pulmonary disease; H, healthy control cohort; ILT, intraluminal thrombus; IQR, interquartile range; P, PAD patient cohort; PAD, peripheral artery disease; n, number of measurements; n.s., not significant. Values are given as median with IQR. Wilcoxon signed rank test was applied. All group differences with p < 0.100 are listed and boldface entries indicate statistical significance.

**Figure 2:**
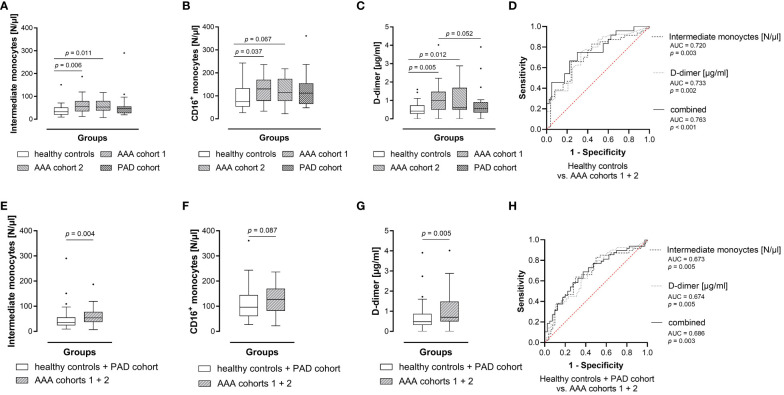
Blood levels of intermediate monocytes hold diagnostic information for AAA. Absolute blood levels of **(A)** intermediate monocytes, **(B)** CD16^+^ monocytes as well as **(C)** plasma D-dimer concentrations between groups. For the diagnostic study, circulating parameter concentrations were measured in blood samples from 25 healthy controls, matched 1:1 (age, sex, best possible in smoking habit) with 25 AAA cohort 1 patients, 22 AAA cohort 2 patients and 25 PAD patients (additionally matched with AAA patients for previous cardiovascular disease). Statistical analysis was performed by Wilcoxon signed rank test, and the boxplots illustrate the parameter values according to Tukey’s style. Only *p* values < 0.100 are shown. Absolute blood levels of **(E)** intermediate monocytes, **(F)** CD16^+^ monocytes as well as **(G)** plasma D-dimer concentrations between combined groups of healthy controls as well as patients with other atheroocclusive disease (PAD), and combined AAA cohorts (1 and 2). Statistical analysis was performed by Mann-Whitney U test, and the boxplots illustrate the parameter values according to Tukey’s style. Only *p* values < 0.100 are shown. The **(D, H)** diagnostic marker value of absolute blood levels of intermediate monocytes or plasma D-dimer, and of the combined parameters was further assessed by receiver operating characteristic (ROC) analyses and the area under the ROC curve (AUC). AAA, abdominal aortic aneurysm; PAD, peripheral artery disease.

Regarding the circulating subsets, intermediate monocytes were significantly elevated in both AAA patient cohorts compared to the healthy controls. We observed a 1.8-fold increase of median intermediate monocyte levels in AAA patient cohort 1 (56 vs 32 N/μl, *p* = 0.006) and a 1.7-fold elevation in AAA patient cohort 2 (53 vs 32 N/μl, *p* = 0.011) compared to the healthy control group (**Figure 2A**). In addition, the combined blood concentration of intermediate and non-classical monocytes, i.e. of circulating CD16^+^ monocytes, was found to be significantly increased in AAA patient cohort 1 (130 vs 75 N/μl, *p* = 0.037) and was borderline increased in AAA patient cohort 2 (115 vs 75 N/μl, *p* = 0.067) when compared to the healthy controls (**Figure 2B**). Interestingly, we also observed a significantly lower level of classical monocytes in healthy individuals, when compared to AAA patients of cohort 2 (396 vs 430 N/μl, *p* = 0.020). Absolute differences in these explorative blood parameters between the groups were also reflected in relative blood concentrations of circulating monocytes: the median percentage of the intermediate subset within monocytes of AAA patient cohort 1 was significantly increased compared to healthy individuals (9.43 vs 6.99%, *p* = 0.004), but also compared to PAD patients (9.43 vs 7.21%, *p* = 0.048). Additionally, the frequency of intermediate monocytes in AAA patient cohort 2 tended to be higher than in healthy controls (8.21 vs 6.99%, *p* = 0.095).

Monocytes were further analyzed for the proportion of monocyte-platelet aggregates, which did not show significant differences in absolute or relative measurements between compared groups.

Concerning the currently best characterized blood-based AAA biomarker, D-dimer (**Figure 2C**), we found a significant elevation of plasma concentration in the AAA patient cohorts (1 and 2) compared to the healthy control collective (1.00 vs 0.43 μg/ml, *p* = 0.005 and 0.60 vs 0.43 μg/ml, *p* = 0.012, respectively). The plasma D-dimer level of AAA cohort 1 was also borderline increased when compared to PAD patients (1.00 vs 0.56 μg/ml, *p* = 0.052).

In ROC analysis (**Figure 2D**) the absolute levels of intermediate monocytes significantly discriminated between AAA patients (combined AAA patient cohorts 1 and 2) and healthy individuals (AUC = 0.720, *p* = 0.003). Likewise, plasma D-dimer concentrations identified AAA patients (AUC = 0.733, *p* = 0.002). When combining both parameters a superior ROC score was achieved in discriminating AAA patients from healthy individuals (AUC = 0.763, *p* < 0.001).

We then proceeded to merge the control groups (healthy controls and PAD cohort) versus AAA (cohorts 1 and 2) to assess whether a blood parameter is effective in detecting AAA against both healthy and CVD background. We identified a 1.5-fold increase in median levels of absolute intermediate monocyte counts in AAA patients (combined AAA cohort 1 and 2) compared to healthy controls or patients with PAD (54 vs 35 N/μl, *p* = 0.004) (**Figure 2E**). In addition, we found that the percentage of intermediate monocytes in AAA patients was significantly elevated compared to healthy individuals or patients with PAD (median 9.16 vs 7.05%, *p* = 0.004). Furthermore, the absolute concentration of circulating CD16^+^ monocytes was borderline increased in AAA patients (127 vs 96 N/μl, *p* = 0.087), when compared to the combined healthy control and PAD collectives (**Figure 2F**). Regarding D-dimer (**Figure 2G**) we observed a significant elevation of plasma concentration in the AAA collective when compared to the combined healthy control and PAD collectives (0.70 vs 0.49 μg/ml, *p* = 0.005). In ROC analysis, circulating absolute levels of intermediate monocytes significantly discriminated between AAA patients (combined AAA patient cohorts 1 and 2) and patients with PAD or healthy individuals (AUC = 0.673, *p* = 0.005) (**Figure 2H**). Likewise, plasma concentrations of D-dimer significantly differentiated individuals with AAA from individuals without aneurysmal disease (AUC = 0.674, *p* = 0.005). The combination of both variables resulted in a higher ROC score (AUC = 0.686, *p* = 0.003).

### 3.3 Circulating monocyte subsets and monocyte-platelet aggregates are associated with AAA progression

**Figure 3:**
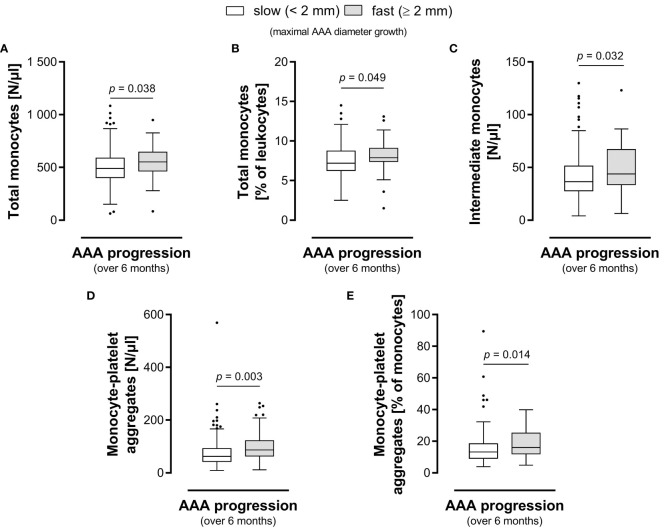
Circulating monocyte-platelet aggregates and intermediate monocytes hold prognostic information for AAA. Group comparison of patients with slow AAA (< 2 mm) versus fast AAA progression (≥ 2 mm) in maximum diameter growth over the next six months. The **(A)** absolute and **(B)** relative blood levels of total circulating monocytes, the **(C)** absolute blood levels of intermediate monocytes, the **(D)** absolute and **(E)** relative blood levels of monocyte-platelet aggregates are shown. Statistical analysis was performed by Mann-Whitney U test, and the boxplots illustrate the parameter values according to Tukey’s style. AAA, abdominal aortic aneurysm.

We further evaluated the biomarker potential of circulating monocyte subsets and monocyte-platelet aggregates to predict AAA growth over the next six or twelve months (**Table 4**). We found a significant prognostic value for the absolute and relative frequencies of circulating total monocytes as well as monocyte-platelet aggregates in predicting fast (≥ 2 mm) AAA progression over a 6-month period. The median absolute (**Figure 3A**) and relative (**Figure 3B**) levels of circulating total monocytes (552 vs 490 N/μl, *p* = 0.038 and 7.90 vs 7.20%, *p* = 0.049) and the median absolute (**Figure 3D**) and relative (**Figure 3E**) levels of monocyte-platelet aggregates (87 vs 63 N/μl, *p* = 0.003 and 16.07 vs 13.20%, *p* = 0.014, respectively) were significantly higher in patients with fast AAA progression over six months. Moreover, the absolute blood concentration of circulating intermediate monocytes (44 vs 36 N/μl, *p* = 0.032) in patients with fast progressing AAA (≥ 2 mm in 6 months) was significantly elevated compared to the levels in slow progressing AAA patients (**Figure 3C**). Additionally, we observed a trend of increased classical monocyte concentrations (420 vs 379 N/μl, *p* = 0.066) in patients with fast AAA progression over the next six months.

**Table 4:** Comparison of baseline blood parameters of patients with slow AAA (< 2 mm) versus fast AAA progression (≥ 2 mm) over six months, and slow AAA (< 4 mm) versus fast AAA progression (≥ 4 mm) over twelve months.

*Parameter*	*Slow progression*		*Fast progression*		*p value*
*Six months AAA progression*	n	Median (IQR)	n	Median (IQR)	
*Total monocytes [N/μl]*	161	490 (187)	37	552 (181)	**0.038**
*Classical monocytes [N/μl]*	157	379 (153)	35	420 (189)	0.066
*Intermediate monocytes [N/μl]*	157	36 (24)	35	44 (34)	**0.032**
*Non-classical monocytes [N/μl]*	157	59 (35)	35	59 (32)	0.668
*CD16^+^ monocytes [N/μl]*	157	96 (48)	35	103 (55)	0.203
*Monocyte-platelet aggregates [N/μl]*	157	63 (50)	35	87 (60)	**0.003**
*Total monocytes [% of leukocytes]*	161	7.20 (2.50)	37	7.90 (1.70)	**0.049**
*Classical monocytes [% of monocytes]*	157	79.15 (6.62)	35	81.44 (10.46)	0.661
*Intermediate monocytes [% of monocytes]*	157	8.08 (3.15)	35	8.67 (5.01)	0.234
*Non-classical monocytes [% of monocytes]*	157	12.53 (6.26)	35	12.07 (5.75)	0.339
*CD16^+^ monocytes [% of monocytes]*	157	20.85 (6.62)	35	18.56 (10.46)	0.661
*Monocyte-platelet aggregates [% of monocytes]*	157	13.20 (9.36)	35	16.07 (13.33)	**0.014**
*D-dimer [μg/ml]*	148	0.80 (0.70)	26	0.92 (1.30)	0.190
*Twelve months AAA progression*					
*Total monocytes [N/μl]*	130	491 (207)	18	552 (165)	0.096
*Classical monocytes [N/μl]*	128	385 (170)	17	429 (111)	0.156
*Intermediate monocytes [N/μl]*	128	39 (30)	17	44 (41)	0.261
*Non-classical monocytes [N/μl]*	128	55 (37)	17	65 (42)	0.212
*CD16^+^ monocytes [N/μl]*	128	96 (50)	17	112 (70)	0.240
*Monocyte-platelet aggregates [N/μl]*	128	65 (52)	17	87 (109)	0.052
*Total monocytes [% of leukocytes]*	130	7.20 (2.63)	18	8.10 (1.83)	0.053
*Classical monocytes [% of monocytes]*	128	79.53 (7.60)	17	81.84 (8.60)	0.830
*Intermediate monocytes [% of monocytes]*	128	8.23 (3.32)	17	8.02 (4.27)	0.931
*Non-classical monocytes [% of monocytes]*	128	12.18 (6.58)	17	12.07 (5.84)	0.951
*CD16^+^ monocytes [% of monocytes]*	128	20.47 (7.60)	17	18.16 (8.61)	0.830
*Monocyte-platelet aggregates [% of monocytes]*	128	13.25 (10.01)	17	14.78 (15.92)	0.269
*D-dimer [μg/ml]*	113	0.77 (0.71)	12	0.90 (0.58)	0.361

AAA, abdominal aortic aneurysm; IQR, interquartile range; n, number of measurements. Values are given as absolute or relative median with IQR. Mann-Whitney U test was applied. Boldface entries indicate statistical significance.

There was no significant prognostic value for any investigated parameter in predicting fast (≥ 4 mm) AAA progression over a 12-month period. Yet, we found borderline elevated levels of absolute and relative frequencies of total circulating monocytes (552 vs 491 N/μl, *p* = 0.096 and 8.10 vs 7.20%, *p* = 0.053) as well as borderline increased absolute concentrations of monocyte-platelet aggregates (87 vs 65 N/μl, *p* = 0.052) in patients with fast versus slow AAA progression over a 12-month period.

The median plasma levels of D-dimer were comparable between patients with slow and fast progressing AAA over both the next six and twelve months (0.80 vs 0.92 μg/ml, *p* = 0.190 and 0.77 vs 0.90 μg/ml, *p* = 0.361, respectively).

The parameter of highest significance, the absolute blood level of monocyte-platelet aggregates, was further evaluated by univariable and multivariable binary logistic regression analysis to identify fast AAA progressors over 6 months. Following the adjustment for morphological and blood parameters as metric variables as well as comorbidities and patient medication as categorical variables, the total blood level of monocyte-platelet aggregates (*p* = 0.038) held independent prognostic information for AAA growth (**Table 5**).

**Table 5:** Multivariable analysis (binary logistic regression) of AAA progression (over the next six months).

*Parameter*	*Odds ratio*	*95% CI* *lower value*	*95% CI* *upper value*	*p value*
*Monocyte-platelet aggregates [N/µl]*	1.006	1.000	1.011	**0.038**
*Maximum AAA diameter [mm] at first visit*	1.077	1.017	1.141	**0.011**
*Age [years] at first visit*	0.957	0.902	1.014	0.136
*Sex [male]*	0.314	0.109	0.907	**0.032**
*Nicotine current*	1.619	0.663	3.954	0.659
*Hypertension*	1.729	0.411	7.281	0.455
*Hyperlipidemia*	1.700	0.669	4.321	0.265
*Constant*	0.153			0.426

AAA, abdominal aortic aneurysm; CI, confidence interval. Boldface entries indicate statistical significance.

Since serial 6-months and 12-months monitoring periods were derived from the same 60 patients, we introduced another variable to account for multiple measurements in AAA patients over time. In order to approximate an average growth in maximum aortic diameter over the entire monitoring period, we calculated a linear slope of AAA progression (k) and found significant positive correlations (**Table 6**) with the absolute blood concentrations of circulating total monocytes (R = 0.280, *p* = 0.032), intermediate monocytes (R = 0.353, *p* = 0.010), and monocyte-platelet aggregates (R = 0.403, p = 0.003) at baseline.

**Table 6:** Correlation of baseline blood parameters with AAA progression over the entire monitoring period (linear slope k of AAA growth).

*Parameter*	*n*	*Spearman’s rho*	*p value*
*Total monocytes [N/μl]*	59	0.280	**0.032**
*Total monocytes [% of leukocytes]*	59	0.222	0.090
*Intermediate monocytes [N/μl]*	53	0.353	**0.010**
*Intermediate monocytes [% of monocytes]*	53	0.147	0.294
*Monocyte-platelet aggregates [N/μl]*	53	0.403	**0.003**
*Monocyte-platelet aggregates [% of monocytes]*	53	0.197	0.157
*D-dimer [μg/ml]*	44	0.005	0.973

AAA, abdominal aortic aneurysm; n, number of measurements. For analyses of correlations, the Spearman’s rank correlation coefficient (Spearmans’s rho) was calculated. Boldface entries indicate statistical significance.

## 4 Discussion

The potential of circulating monocyte subsets as biomarkers for human AAA has not been extensively investigated. This study evaluated circulating monocyte subsets as well as MPA and their association with AAA disease state and progression, to determine their diagnostic and prognostic marker value. We found that the blood count of total monocytes, but especially the levels of the intermediate subset and combined CD16^+^ monocytes (intermediate and non-classical monocytes) are elevated in AAA patients compared to healthy controls. In line with our own previous data and the evidence of other studies, the CD16^+^ monocytes show an increase in acute and chronic inflammatory conditions (22, 45). Similarly, it has previously been reported that AAA patients exhibit a higher percentage of circulating intermediate and non-classical monocytes compared to healthy controls (21, 33). Compared to the results of Ghigliotti et al. (21), the AAA patient population in this study presented slightly higher frequencies of intermediate monocytes (AAA cohort 1: 9.4%, AAA cohort 2: 8.2% versus 7.7% in Ghigliotti´s analysis). Yet, also the frequency of intermediate monocytes in the healthy collective of our study was higher (7.0%) while it ranged at 5.4% in the study of Ghigliotti et al. (21). These differences may relate to distinct immunostaining and flow cytometry procedures, since Ghigliotti et al. conducted mononuclear cell isolation and CD56+ cell exclusion, while our study was based on whole blood immunostaining and CD14/HLA-DR monocyte identification. Data on the non-classical monocyte subset in AAA are controversial. Ghigliotti et al. reported an elevation of these cells in circulation of AAA patients, while Rubio-Navarro et al. did not detect this increase (21, 33). Our own data support the notion that non-classical monocytes do not significantly contribute to the rise in CD16+ monocytes in AAA patients.

Regarding PAD patients, none of the investigated monocyte subsets were significantly elevated compared to healthy controls. In contrast, a study including 143 patients with PAD observed significantly increased intermediate monocytes and demonstrated that the rise in frequencies of intermediate monocytes occurs with more advanced, late stages of PAD (Rutherford stage IV-VI) (46). In this study, the PAD collective was categorized with 48% stage II and 12% stage III, representing a less severe disease cohort. This has to be taken into account with respect to our observation that AAA patients (AAA cohort 1: 9.4%, AAA cohort 2: 8.2%) showed a higher frequency of intermediate monocytes than the PAD cohort (7.2%).

Importantly, the circulating absolute levels of intermediate monocytes in this study showed potential for the discrimination of AAA patients from a combined non-AAA collective of healthy individuals and PAD patients, thus reaching the biomarker value of D-dimer. Combining the two parameters resulted in a higher marker potential than achieved for either variable separately. This may be partly due to the fact that D-dimer levels represent the pathogenic component of hemostasis/fibrinolysis while monocytes reflect the chronic inflammatory process at the AAA site.

Surprisingly, MPA were not significantly elevated in AAA patients versus control groups. Yet, they presented sensitive in indicating the course of disease. Thus, the blood count of total monocytes, of the intermediate subset and of MPA predicted fast AAA progression over a short-term period of six months, while a more long-term prognostic marker potential over twelve months was not given. Notably, several studies emphasized the important role of MPA in cardiovascular conditions (47). Especially thromboembolic events in the context of coronary heart disease, unstable angina pectoris, atrial fibrillation or myocardial infarction seem to be associated with elevated blood levels of MPA (48–50). Thus, rather than being specific or suited as a diagnostic tool for AAA detection, MPA may assist to predict the inflammatory-driven disease progression in longitudinal monitoring. Interestingly, it is possible that the elevation of MPA is connected to the higher intermediate monocyte levels in AAA patients, because it was shown that intermediate and non-classical monocytes more readily form aggregates with platelets than classical monocytes do (35). While our analysis predominantly focused on defined monitoring periods of 6 or 12 months, it was remarkable that baseline levels of intermediate monocytes and MPA also correlated significantly with the entire course of a patient´s disease as reflected in the calculated growth slope. In multivariable analysis blood levels of MPA prevailed, i.e. were independently associated with faster AAA progression over six months, along with starting diameter and female sex.

Regarding study restrictions, the data were evaluated retrospectively and the sample size of our study, despite being based on carefully matched collectives, certainly limits the assessment of the diagnostic and prognostic marker potential. The study was explorative in design, so no adjustments for multiplicity of errors were made. The strengths of the study are the very coherent 1:1 matched cohorts, precisely defined time points for blood sampling and CTA analyses. In particular, the assessment of AAA progression via CTA at 6-month intervals represents a highly sensitive method of determining aneurysm growth compared to sonography as performed in many other studies. Of note, the study design was unusual with respect to the inclusion of two AAA cohorts which was due to the endeavor of a careful 1:1 matching with PAD patients (including prior cardiovascular events). The fact that not all findings were consistent, i.e. a number of findings differed between the two AAA cohorts when compared to controls, indicates that - despite careful matching - unrecognized factors may have affected results in one of two AAA study groups.

## 5 Conclusion

This single-center retrospective cohort study supports the evidence that circulating monocyte subsets are elevated in AAA patients. The combination of biomarkers for inflammation (intermediate monocytes) and hemostasis (D-dimer) may prove particularly beneficial in AAA diagnosis. Furthermore, our study provides first evidence for the prognostic value of intermediate monocytes and MPA in AAA; MPA were identified as independent predictor of AAA progression in multivariable analysis. Prognostic AAA biomarkers are particularly desired since to date the maximum aortic diameter is the sole clinically applied parameter to predict AAA growth. Thus, large-scale prospective studies are warranted to validate these biomarkers which may hold potential for timing and decision guidance of clinicians for diagnostic and therapeutic interventions.

## Data availability statement

The raw data supporting the conclusions of this article will be made available by the authors upon reasonable request to the corresponding author.

## Ethics statement

The studies involving humans were approved by the institutional ethics committee of the Medical University of Vienna. The studies were conducted in accordance with the local legislation and institutional requirements. The participants provided their written informed consent to participate in this study.

## Author contributions

JK: Conceptualization, Data curation, Formal analysis, Investigation, Methodology, Validation, Visualization, Writing – original draft, Writing – review & editing. BZ: Conceptualization, Data curation, Investigation, Methodology, Writing – review & editing. AB: Data curation, Formal analysis, Investigation, Writing – review & editing. PL: Data curation, Formal analysis, Investigation, Writing – review & editing. CC: Data curation, Formal analysis, Investigation, Writing – review & editing. PR: Data curation, Formal analysis, Investigation, Writing – review & editing. NC: Data curation, Formal analysis, Investigation, Writing – review & editing. MZ: Data curation, Formal analysis, Investigation, Writing – review & editing. LF: Data curation, Formal analysis, Investigation, Writing – review & editing. HH: Conceptualization, Methodology, Writing – review & editing. CK: Funding acquisition, Writing – review & editing. WE: Conceptualization, Data curation, Formal analysis, Funding acquisition, Investigation, Methodology, Writing – review & editing. CN: Conceptualization, Funding acquisition, Methodology, Resources, Writing – review & editing. CB: Conceptualization, Data curation, Formal analysis, Funding acquisition, Investigation, Methodology, Writing – original draft, Writing – review & editing.
