# Supplementary material for: Circulating monocyte populations as biomarker for abdominal aortic aneurysms: a single-center retrospective cohort study
Source: Front Immunol. 2024 Jul 30;15:1418625. doi: 10.3389/fimmu.2024.1418625 (PMC11319298; doi:10.3389/fimmu.2024.1418625)
Supplement: Supplementary file 1 [file DataSheet_1.pdf]

Supplementary Material

Circulating monocyte populations as biomarker for abdominal aortic aneurysms: a single-center retrospective cohort study

Johannes Klopf[1], Branislav Zagrapan[1], Annika Brandau[1], Peter Lechenauer[1], Catharina J. Candussi[1], Patrick Rossi[1], Nihan Dide Celem[1], Michael Ziegler[1], Lukas Fuchs[1], Hubert Hayden[1], Claus G. Krenn[2], Wolf Eilenberg[1], Christoph Neumayer[1], Christine Brostjan[1*]

[1] Division of Vascular Surgery, Department of General Surgery, University Hospital Vienna, Medical University of Vienna, Vienna, Austria

[2] Intensive Care Medicine and Pain Medicine, Department of Anesthesia, University Hospital Vienna, Medical University of Vienna, Vienna, Austria

*** Correspondence:** Christine Brostjan

Division of Vascular Surgery, Department of General Surgery, Medical University of Vienna, University Hospital Vienna, Waehringer Guertel 18-20, A-1090, Vienna, Austria

Telephone: +43-(0)1-40400-73514

Fax: +43-(0)1 40400-73593

E-mail: christine.brostjan@meduniwien.ac.at

1 Supplementary Methods

Flow cytometry of monocyte populations

Peripheral venous blood was collected in hirudin tubes (Roche Diagnostics, Mannheim, Germany) and immediately fixed with ThromboFix Platelet Stabilizer solution (Beckman Coulter, Brea, California, U.S.A., no. 6607130). Whole blood samples were incubated at room temperature for 2 to 5 hours and monocyte subsets were immunostained using the following antibodies: FITC conjugated anti-human CD14 antibody (BioLegend, San Diego, California, U.S.A., clone HCD14, no. 325604); PE labeled anti-human HLA-DR antibody (BioLegend, clone L243, no. 307606); PE/Cy7 stained anti-human CD16 antibody (BioLegend, clone 3G8, no. 302016); Pacific Blue labeled anti-human CD41 antibody (BioLegend, clone HIP8, order no. 303714). To exclude nonviable cells from the analysis, 7-amino-actinomycin D staining (7-AAD, Immunotech, Beckman Coulter, no. A07704) was applied. After erythrocyte lysis with RBC Lysis Buffer (Thermo Fisher Scientific, Waltham, Massachusetts, U.S.A., no. 00-4333-57), the samples were diluted with Dulbecco's phosphate buffered saline with $CaCl_2$ and $MgCl_2$ and immediately analyzed with a Gallios flow cytometer (Beckman Coulter) equipped with Kaluza for Gallios software (version 1.1.20388.18228, Beckman Coulter). For the distinction between classical ($CD14^{++}CD16^{-}$), intermediate ($CD14^{++}CD16^{+}$) and non-classical ($CD14^{+}CD16^{++}$) monocytes, the subsets were quantified from viable $HLA\text{-}DR^{+}CD14^{+}$ cells according to their cell surface expression of CD14 and CD16 (**Supplementary Figure 1**). Monocytes were analyzed for the proportion of monocyte-platelet aggregates, which were identified via CD14 and CD41 signal. A complete blood count was performed for the same hirudin-treated blood sample on a Sysmex XN-350 hemocytometer (Sysmex, Kobe, Japan), which was required for calculations of absolute leukocyte subset concentrations. Flow cytometric analyses were performed with Kaluza software (version 1.3, Beckman Coulter) by investigators blinded to study groups. In addition, peripheral venous blood was processed for measurement of routine laboratory parameters.

2 Supplementary Figures

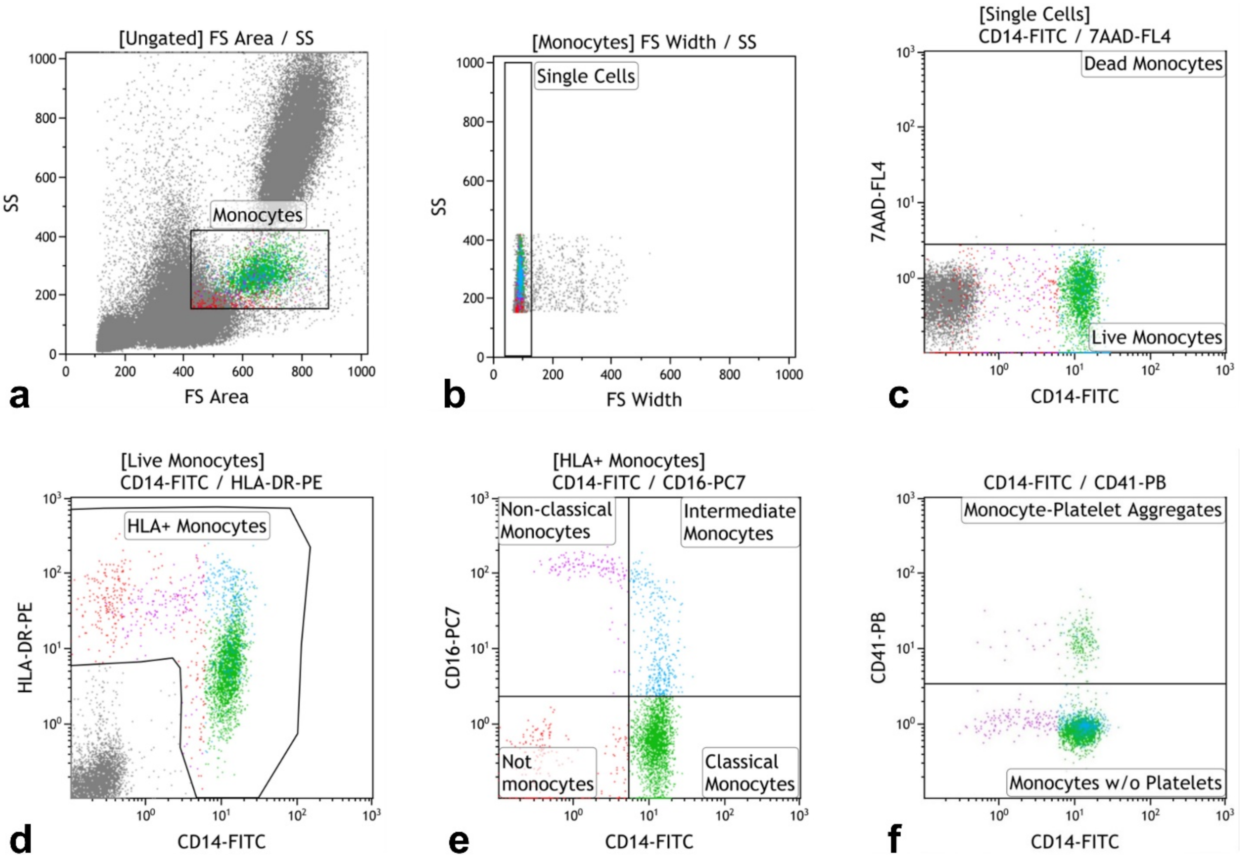

Supplementary Figure 1. Gating strategy for monocyte subsets. **(a)** Monocytes were identified in a forward scatter / side scatter dot plot to roughly discriminate them from lymphocytes and granulocytes. **(b)** Next, single cells were selected based on forward scatter width and **(c)** dead cells were excluded by 7AAD signal. After gating for **(d)** viable HLA-DR^{+}CD14^{+} cells, the subsets of **(e)** classical (CD14^{++}CD16^{-}), intermediate (CD14^{++}CD16^{+}) and non-classical (CD14^{+}CD16^{++}) monocytes were quantified according to their cell surface expression of CD14 and CD16. **(f)** Monocytes were analyzed for the proportion of monocyte-platelet aggregates, which were identified via co-signal of CD14 and CD41.
